# Methodological issues of confounding in analytical epidemiologic studies 

**Published:** 2012

**Authors:** Karimollah Hajian Tilaki

**Affiliations:** 1 Department of Social Medicine and Health, Babol University of Medical Sciences, Babol, Iran.

**Keywords:** Confounding, Bias, Observational studies, Non-randomized experimental studies, Risk ratio, Statistical models, Adjustment

## Abstract

Confounding can be thought of as mixing the effect of exposure on the risk of disease with a third factor which distorts the measure of association such as risk ratio or odds ratio. This bias arises because of complex functional relationship of confounder with both exposure and disease (outcome). In this article, we provided a conceptual framework review of confounding issues in epidemiologic studies, in particular in observational studies and nonrandomized experimental studies. We have shown in 2 by 2 tables with analytical examples how the index of association will be distorted when confounding is present. The criteria, source of confounding and several points in confounding issues have been addressed. The advantages and disadvantages of several strategies for control of confounding have been discussed.

Many epidemiologic studies are planned to examine the causal association of exposure with the outcome of interest using non-experimental (observational) or experimental data (1). By definition, for judging causal inference, the following three criteria simultaneously should be satisfied (2): 1) exposure must be proceeded from outcome (temporal sequence); 2) a statistical association should be revealed between exposure and outcome i.e. any changes on exposure status yields changes on outcome; 3) the apparent association must be valid. It means that the derived association should not result from any systematic errors such as confounding, information bias, selection bias and random errors as well. The strength of association between exposure and outcome depends on the magnitude of risk ratio (RR) or odds ratio (OR) that is revealed in the study. Confounding is the main issue in observational etiologic studies and non-randomized interventional studies as well (3-5). In the context of epidemiology, confounding is a source of bias in estimating causal association and it corresponds to a lack of comparability between the exposed and non-exposed groups (or cases and controls) (6). In this context, confounding is described as a mixing of extraneous factor (called confounder) with the effect of exposure of interest (5, 6). This happens in epidemiologic studies with non-experimental data. In the context of biostatistics, confounding refers to magnitude of an association parameter estimation with respect to adjusting versus not adjusting for extraneous variables (called confounding) (2, 6, 7). The presence of imbalance covariate (that is called confounder) between the compared groups (e.g. exposed and non-exposed or cases and controls) distorts the association of interest if one does not take it into account in design or analysis. The mixing effects of exposure and confounder result from a complexity of inter relationship of confounder with exposure and outcome as well (5). 

In observational studies, there is always a possibility to influence such extraneous variables on the outcome of interest because of lack of comparability of two groups at baseline (6, 8, 9). In randomized clinical trials (RCT), causal inference emphasizes the importance of randomization in assuring the comparability while in an observational study no such assurance is available thus, the issues of confounding become predominate (6). This article provided a conceptual framework of review of methodological issues on the role of confounding in analytical epidemiologic studies.

## Methodology

**Definition of Confounding**

Mixing the effect of exposure (or treatment) on occurrence of disease (or outcome) with a third factor (called confounder) happens when the third factor is an independent risk factor for disease and also it should have association with exposure independently (4, 5). Such a covariate should not be at intermediate pathway relation between exposure and disease (5). Depending on the inter-relation between confounder with exposure and outcome, the lack of its control is resulted as over or under estimate of measure of association. Complexity of confounding variable has arisen via two directional associations of confounder with exposure and outcome. By definition, the three conditions should be met for a factor to be confounder. i) Confounder factor should associate with exposure i.e. it should have imbalance distribution between the exposed and the non-exposed groups. For example, age is a cofounder if the distribution of age differs between exposed and non-exposed groups. ii) confounding variable should be an independent risk factor for disease (or outcome) of interest. This inherent association must be present in both the exposed and the non-exposed groups. iii) The association of confounder and disease (or outcome) should not be resulted via exposure (5) i.e. this association should not be an intermediate pathway relation between exposure and outcome. If any of these three conditions is not satisfied, the mixing effect with exposure will not occur and the third variable is no longer a confounder.

**Examples of Mixing Effect with Confounding**

Let us assume a cohort study was designed to determine the association between physical activity and myocardial infarction (MI). Age can be considered as a confounder that distorts the magnitude of this association since the distribution of age may differ between those with and without physical activities and the group with physical activities may be younger thus, age is associated physically (condition 1). The younger subjects may have a lower risk of MI for both with and without physical activities. Thus, age is inherently a risk factor for MI (condition 2). In addition, the effect of age is not on a pathway relation of physical activities and MI (condition 3). So, the lack of control of the effect of age, the apparent association between physical activities and MI is confounded by age. In this example, the question will arise in the distortion of this association to which direction occurs. Since the physical active group is younger and the younger people has a lower risk of MI, consequently, the mixing effect of age with physical activities leads to the exaggeration of the inverse association between physical activities and MI. In this example, we call age as a positive confounder. In the context of this example, gender also can be considered as confounding since the level of physical activities differs between men and women, and men have a greater risk for MI both with and without physical activities. The lack of control for gender leads to the dilution of the inverse association between physical activity and MI. Thus, gender is a negative confounder. In analytical epidemiologic studies, age and gender are associated with several lifestyles, physical and chemical exposures and they are also risk factors for disease (or outcome) of interest. Thus, their rules as confounder variables should be taken into account. 

**Crude and Specific Effect: Examples of 2 by 2 Tables**

In this section, with several hypothetical examples, we have shown how collapsing data over different stratum of confounding factor can distort the risk ratio in analysis 2 by 2 tables.

Example 1. We have shown an example that the lack of control for confounding (collapsing data in a single 2 by 2 table) produces a false association in table 1. For example, in a cohort study, an investigator wishes to determine the association between consumption of vitamin and depression, the collapsing data over the different stratum of covariate of ages is shown in table 1.

**Table 1:** Association of vitamin and depression in 2×2 table

**Depression**	**Vitamin**	
	**+**	**-**
+ -	260 840	1220 880
Total	1100	2100

$\mathrm{Crude RR}=\frac{260}{1100}:\frac{1220}{2100}=0.41$

**Table 1.a:** Association of vitamin and depression among youth in 2 ×2 table

**Depression**	**Vitamin**	
	**+**	**-**
+ -	200 800	20 80
Total	1000	100

$\mathrm{Age specific RR}=\frac{200}{1000}:\frac{20}{100}=1$

**Table 1.b:** Association of vitamin and depression among old in 2 ×2 table

**Depression**	** Vitamin**	
	**+**	**-**
+ -	60 40	1200 800
Total	100	2000

$\mathrm{Age specific RR}=\frac{60}{100}:\frac{1200}{2000}=1$

 As table 1 shows, the calculated crude estimate of RR=0.41 revealed that vitamin reduced the risk of depression about 59%. While in tables 1.a and 1.b, when data are stratified with respect to age group, the age specific effect of vitamin on depression does not longer appear. For both young and old people, the age specific RR is equal to 1 that shows there is no association between vitamin D and depression. Thus, the crude estimate of protective effect of vitamin D on depression based on collapsing data is confounded by age. Regarding to the definition of confounding, one can see whether the confounding criteria are present in these data. In young subjects, $\frac{1000}{1100}=90\%$ were vitamin users while for the old people, there were$\frac{100}{2100}=4.2\%$. Therefore, age is associated with consumption of vitamin (condition1). Also, age is a risk factor for depression among with and without vitamin users. Tables 2 shows that among the vitamin users, the incidence rate of depression is $\frac{200}{1000}=20\%$ and $\frac{60}{100}=60\%$ in young and old people, respectively. Also, among the non-users, the risk of depression is $\frac{20}{100}=20\%$ and $\frac{1200}{2000}=60\%$ in young and old people, respectively. Thus, in both groups, the old people have higher risk for depression (condition 2). Obviously, age effect was not in an intermediate pathway relationship between vitamin consumption and depression (condition 3).

Example 2. This example shows that confounder partially distorts the association. For example, in a cohort study, the effect of smoking on occurrence of heart disease, the results of crude data (collapsing on stratum of confounder) is shown in table 2.

**Table 2:** Association of smoking and heart disease in 2 ×2 table

**Heart disease**	** Smoking**	
	**+**	**-**
+ -	200 800	50 950
Total	1000	1000

$\mathrm{RP}=\frac{200}{1000}:\frac{50}{1000}=4$

**Table 2.a:** Association of smoking and heart disease in patients with diet in 2×2 table

**Heart disease**	** Smoking**	
	**+**	**-**
+ -	194 706	21 79
Total	900	100

$\mathrm{Diet specific RR}=\frac{194}{900}:\frac{21}{100}=1.03$

**Table 2.b:** Association of smoking and heart disease in patients without diet in 2×2 table

**Heart disease**	** Smoking**	
	**+**	**-**
+ -	6 94	29 871
Total	100	900

$\mathrm{Diet pecific RR}=\frac{6}{100}:\frac{29}{900}=1.86$

As table 2 shows, the crude estimate of RR was 4. When data are stratified based on diet status, the results are presented in tables 2.a and 2.b for with and without diet respectively. The diet specific RR was 1.03 for diet users and 1.86 for non-users. One can see easily that the crude estimate of RR lies out of the range of Specific RR. Thus, diet is a confounder factor and lack of its control, the positive association between smoking and risk of heart disease is exaggerated. In this example, the diet is not only a confounder but also a modifier as well. Since the effect of smoking is quite different in two levels of diet status.

Example 3. This example shows that the mixing effect of confounder with exposure distorts the real effect toward the null value i.e. the real effect will be diluted by confounding. In a cohort study, to determine the association between air pollution and pulmonary disease the results of collapsing data were presented in table 3.

**Table 3:** Association of air pollution and pulmonary disease in 2 ×2 table

**Pulmonary disease**	** Air pollution**	
	**+**	**+**
+ -	200 1800	400 3600
Total	2000	4000

$\mathrm{RR}=\frac{200}{2000}:\frac{400}{4000}=1$

**Table 3.a:** Association of air pollution and pulmonary disease among women in 2 ×2 table

**Pulmonary disease**	** Air pollution**	
	**+**	**-**
+ -	110 390	380 2620
Total	1500	3000

$\mathrm{Sex specific RR}=\frac{110}{500}:\frac{380}{3000}=1.74$

**Table 3.b:** Association of air pollution and pulmonary disease among men in 2 ×2 table

**Pulmonary disease**	** Air pollution**	
	**+**	**+**
+ -	90 1410	20 980
Total	1500	1000

$\mathrm{Sex specific RR}=\frac{90}{1500}:\frac{20}{1000}=3$

As table 3 shows the crude RR was 1 that means there is no association between air pollution and pulmonary disease. While the sex specific RR was 1.74 and 3 for women and men, respectively. The crude RR lies out of the range of sex specific RR that shows the lack of control for gender, the effect of interest is diluted toward the null value. Moreover, the sex specific RR for smoking varies from 1.74 to 3, thus, gender should be considered as a modifier as well.

**Several Points on Confounding Characteristics: **We draw the attention of researchers and clinicians to the following points: First, confounder variables must be independently associated with disease (or outcome) of interest (i.e. risk factor for disease) for both the exposed and the non-exposed groups. If a covariate is associated with disease in exposed group not for non-exposed, this association might be triggered by exposure only. If this is the case, this variable is no longer a confounder. For example, if an association between sugar consumption and MI only was present in smokers not for non-smokers, sugar consumption would not be considered as confounding factor. Second, confounding factors must not be an intermediate variable in causal pathway relationship between exposure and disease (or outcome). For example, if the association of high density lipoprotein (HDL) with alcohol consumption and MI was in an intermediate causal pathway between alcohol consumption and MI, then HDL could not be considered as confounding and its effect should not be controlled in statistical analysis (3) since preventing the effect of HDL, the effect of alcohol would be diluted. Third, in practice, if the adjusted effect of OR (or RR) is substantially different with the crude estimates of OR (or RR), then the adjusted covariate is a confounder (2). 

Fourth, the potential effect of confounding is revealed by the magnitude of distortion of association between exposure and disease. The amount of this distortion depends on the magnitude of association of confounder with exposure and disease. Regarding the types of these association (positive or negative), the effect of interest might be over or underestimated (2). Fifth, one should distinguish between confounding and effect modifier, although a confounder factor may have the role of modifier as well, these two concepts are different (4). In analytical studies for prevention of mixing effect, control for confounding is necessary. Lack of its consideration yields invalid results, while lack of revealing the effect modification does not distort overall effects. Sixth, in randomized clinical-trial study, there is less concerned of imbalance characteristics between two groups of comparison with respect to some baseline variables (10, 11) since random allocation prevents imbalance data and thus, confounding is no longer present if sufficient sample size is recruited, in particular, randomization an unknown confounding in an design of study. 

**Sources of confounding: **There are several sources for confounding:

**Susceptibility bias**: This bias occurs, because of the presence of susceptible factors at baseline (before exposure). Thus, the risk of outcome in exposed group is substantially greater than the non-exposed group at baseline (12).

**Exposure selection bias**: This happens when the subjects or their family or their physician select exposure of interest. The motivation of such selection is usually associated with outcome. This could be considered a special case of susceptibility bias. 

For example, in observational cohort study, the behavior of mothers is compared in two groups of breast feeding and bottle feeding, this comparison is prone to be confounded if mothers selected breastfeeding to bottle feeding, differed with respect to some psychological behaviors that might affect the caring of their infants (12).

**Clinical indication**: This bias occurs when an observational study compares the effect of different therapeutic manures. This confounding bias is called confounding by indication (or channeling bias) and it is a serious bias in non-experimental studies of medication effect (6, 11, 13-15) because clinical indication affects on the choice of treatment that is independently associated with outcome. For example, channeling occurs when drug therapies preferentially prescribed to group of patients with specific risk profile (with evidence of clinical indications). Those who may not have such indication are prescribed with an alternative therapeutic agents. In clinical practice, the newer therapy is often assigned to profile of patients that is more likely to get benefit (6, 13). Thus, this leads to the incomparability of prognostic factors and baseline morbidity between the subjects receiving the new therapeutic agent versus the old ones.

**Contaminated bias**: Exposure which is accompanied with other factors (or with other maneuvers) that can affect on outcome (12). This particularly happens in an observational study of therapeutic agents. For example, patients received a new therapeutic agent, probably they receive more care and surveillance as compared with those that receive standard therapeutic. This accompaniment may lead to more events of interest occurring in the first group.

**Confounding in diagnostic studies: **In diagnostic studies which evaluate the accuracy of diagnostic tests versus gold standard, the receiver operator characteristic (ROC) analysis has become a popular method to determine the accuracy of quantitative (or rating) test results and the area under curve (AUC) has been used as measurement of accuracy with meaningful interpretation (16-19). 

Confounding not only distorts the validity of results in association studies, but also it can threaten the validity of diagnostic accuracy derived from the evaluation of new diagnostic test versus a gold standard. Lack of consideration of covariates (confounders) in the design of diagnostic studies or analysis for the evaluation of accuracy of diagnostic test leads accuracy index erroneous distortion (i.e. over or underestimated). This occurs when covariate is associated with both test results and the true state of disease. Janes and Pepe (2011) graphically showed that the overall ROC curve and corresponding AUC substantially differed from stratum specific ROC curve and their AUC when confounding was present (20). 

Thus, the methods for covariate adjustment are required in ROC analysis. For example, for the evaluation of the accuracy of PSA (prostate serum antigen) concentration, the influence of patient age has been revealed (21). Nevertheless, there are a few clinical investigations considered for the adjustment of the effect of covariates in clinical practice of diagnostic test evaluation. The lack of popular method and the availability of software for adjustment and also the lack of awareness of clinicians may be an possible explanation why control for confounding has been rarely used by clinical investigators in the context of diagnostic studies. 

**Strategies for Control of Confounding: **Control for confounding has an important role on the validity of analytical epidemiologic studies and this depends to what extent the confounding variables were considered and correctly measured. It is necessary that researchers have enough evidences of presence of such variables in the stage of study design and to collect their data in order to be able to control them in their analysis.

To avoid confounding is to obtain a reference population for which to be comparable with study group. Such reference population may not be possible in practice. Thus investigators attempt to construct such population based on the study design for control of confounding. Several methods have been proposed in the stage of design and analysis for prevention of confounding.

**Control of Confounding in Design**

**Restriction**

Restriction is an effective approach for prevention of known risk factor of confounding. For example, if gender imbalance confounds study results, restriction on specific gender (e.g. women) would no longer be a confounder. Nonetheless, restriction on many confounding factors can reduce the number of subjects in the study and the generalizability of results as well. In addition, when a factor is restricted in the design, its effect as a risk factor for disease can not be assessed.

**Matching**

Matching refers to applying a restriction in the selection of reference group in which it should have similarities with some characteristics with the study groups. This can be done with individual matching and group matching. For example, gender imbalance is not found, the proportion of women is similar in two compared groups by gender matching. This requires many reference groups of candidates to meet the matching criteria. In addition, matching becomes difficult if one attempts to match reference group and study group with several confounders.

Although, matching reduces confounding bias in epidemiology, the advantage of matching is not only for control of confounding that can be achieved in analysis without matching. While the advantage is to achieve a greater efficiency in terms of amount of information obtained (5, 6). In case control study, the procedure of matching produces similarity in the distribution of exposure between cases and controls. This process itself produces confounding due to matching and thus this attenuates the measure of association if one does not apply conditional analysis with respect to matching factors (or matched analysis) (5, 22). 

Thus, applying matching in study design requires a conditional analysis for validity of results in case-control studies. While such analysis is not required for cohort studies because the procedure of matching differs in the study design, in cohort study, non-exposed and exposed are matched and matching factors are independent from the outcome of interest that might occur sometimes in the future. Moreover, the effect of matching factor can not be assessed as a risk factor for disease (21). The additional drawback of matching is overmatched. This problem occurs when an investigator matches controls and cases with several matching factors, in particular, the matching factors that are highly correlated with exposure.

**Randomization**

Randomized clinical trials (RCT) are the gold standard for establishing causality in clinical research. Randomized treatment allocation prevents imbalance covariates in the study design. To a large extent, it ensures that patients allocated in treatment groups be similar with respect to baseline characteristics. In particular, random allocation deals with confounding which is unknown and it inherently produces comparable groups if sufficient number of subjects is attained in the study (6, 21). However, to prevent imbalance covariate at baseline by randomization is probabilistic and it depends on some conditions. For example, small sample size, and violation of protocol of RCT, noncompliance and loss to follow up may influence the covariate distribution that becomes imbalance (6).

Block randomization (stratified randomization) with respect to covariate has a great assurance to prevent imbalance covariate. This type of randomization can be applied only for explicitly defined covariate not for unknown covariate. For example, if gender is covariate, stratified randomization by gender potentially prevents imbalance gender. A possible solution in dealing with non-compliance problem is to use intention to treat analysis in which the comparison is carried out by treatment assignment rather than treatment received (6).

**Control of Confounding in Analysis**

In observational study, control for all potential confounders may be impossible in design. Thus the investigator should have planned to adjust the effect of different covariates that can be considered as potential confounders in the stage of analysis. For this purpose, it is required that all potential confounders are measured correctly in the design. Obviously, those that were not measured and also unknown confounders could not be adjusted in analysis. Stratification and regression model are the two approaches for the control of confounding in analysis (23). Stratification is the simplest method for adjustment of confounding. For example gender imbalance can not be confounded with stratum specific effect. The method of adjustment of OR (or RR) proposed by Mantel- Haenzel, uses a weighting average of stratum specific effect of OR (or RR) if there is no heterogeneity of effect across stratum (24). 

This method of control for confounding is easy to understand and the data can be shown in 2 by 2 tables; stratum specific effect can easily be compared with crude effect when collapsing data over different strata in a single table as we have shown in our three examples earlier. Moreover, the stratum specific effect can explore whether the covariate is a modifier. Nevertheless, this method can deal with few confounding factors because of paucity of sample size within each cell and thus the statistical inference becomes unreliable. A more powerful method for control of confounding is using multiple regression models that allow us to adjust the effect of several confounding factors simultaneously without loss of information. The development of several statistical software helps the practitioner to adjust simultaneously several confounding variables. For binary outcome, multiple logistic regression models are commonly used for the adjustment of categorical and continuous covariates in epidemiology for both cohort and case-control studies (25). The coefficients of logistic regression model have a meaningful interpretation as the log of OR (or RR) that is commonly used as a measure of association in epidemiologic studies. 

Consequently, the exponential coefficient that corresponds to exposure is the adjusted OR given all potential confounders to be into account in the regression model. For the continuous outcome, the multiple linear regression models are used for adjustment and the regression coefficients represent the adjusted mean difference of binary exposure and the adjusted increment change in mean of outcome for continuous covariate. For the censored data in survival analysis, Cox regression model is a proper method for the adjustment several covariates and the regression coefficients represent the adjusted log hazard ratio (or risk ratio) and their exponential corresponded coefficient of exposure is the adjusted RR in prognostic studies (26).

Although, statistical models are able to smoothen the sparse data and estimate the adjusted effect of interest, nonetheless, no approach can solve sparse data problem properly (6). The lack of enough sample size affects the precision of estimate of regression coefficients and thus the confidence interval for parameters of interest becomes wider and it is also on the power of statistical tests. Thus, the apparent effect does not appear statistically significant. The other limitation of modeling to data is the violation of assumption used in the model that yields bias in regression coefficients and may distort the standard error of regression coefficient and the measures of association as well. The investigators should justify the reality of assumption used in their data where the model could be applied. We definitely recommend this the to clinician that consults with biostatistician in applying the powerful approach of regression modeling for their statistical analysis. In addition, a practical technique for adjusting several confounding at once is by using propensity score (27). This score is the conditional probability of exposure to a treatment given as a set of observed covariates. This procedure has two steps. First, the score is obtained through regression model and then the score is used to adjust the effect of exposure. This procedure also involve, statistical linear model and logistic regression model (13, 23). 

**Limitation for Control of Confounding**

One limitation for the control of confounding in observational studies is that the data of all potential confounders may not be available since these studies usually use the data that have already been available in the patients’ records. Thus, there are unknown confounders that were not measured. Another limitation is that the measurement errors often occur in the collection of data of confounding because of the soft instrument that is applied in data collection. Or even there is a possibility of misclassification for binary confounder. Therefore, the residual confounding (i.e. uncontrolled confounding) almost threatens the study results in observational studies (28-30). In addition, the lack of overlap information regarding confounding factors between the two groups of comparisons makes it difficult using the statistical model for adjustment.

## Conclusion

The proper measuring of potential confounding factors besides exposure and outcome in study design helps the statistician to control them in analysis. In some situations, in particular confounding by indication in non- randomized interventional studies, measuring for indication variables for a particular drug therapy as a proxy and adjusting in analysis help in a way that such confounding is partially adjusted. A proper control for confounding bias does not assure the validity of the study unless the other sources of bias such as information bias, selection bias and also random errors (chance) are excluded. Additionally, a proper design for assurance of causality inference is necessary.
